# A suicide bereavement model: based on a meta-ethnography of the experiences of adult suicide loss survivors

**DOI:** 10.3389/fpubh.2025.1596961

**Published:** 2025-07-14

**Authors:** John Whitebrook, Caroline Lafarge, Jamie S. Churchyard

**Affiliations:** Department of Psychology, School of Human and Social Sciences, University of West London, Ealing, United Kingdom

**Keywords:** suicide loss survivors, suicide bereavement model, meta-ethnography, qualitative research, postvention

## Abstract

**Introduction:**

The annual suicide death rate is c.760,000 therefore, using the widely accepted estimate of 135 people being exposed to each suicide, the worldwide annual exposure rate is over 100 million. While male suicide-loss survivors (SLSs) are equally exposed, the vast majority of suicide bereavement research includes a large majority of female participants.

**Methods:**

Following the eMERGe and PRISMA guidelines, a meta-ethnography (systematic review of qualitative studies) was carried out to assess historical research into suicide-loss survivorship. Seven data sources were searched, up to 30-Nov-2022, for peer-reviewed studies, written in English, that used identifiable and interpretative qualitative methods, had at least 50% male participation, and offered a valuable contribution to the synthesis.

**Results:**

Overall, 1,645 records were screened, and 15 reports of included studies assessed. Eight main themes were identified: changed forever, trauma, stigmatization, protector, lost futures, lost in plain sight, societal norms, and dualities. Via line of argument synthesis, and considering the broader literature, a model for suicide bereavement, applicable to all, is proposed that brings together the gamut of pertinent factors into an integrated framework.

**Discussion:**

The model could be used in practice (clinical, therapy/counseling, education) to enable better understanding of the highly complex and interwoven components of suicide bereavement, thereby facilitating improved and extended services available to SLSs that are more in-tune with their needs. While the model cannot confer full comprehension of suicide bereavement, it can go a long way to assist those looking to assist SLSs by providing a platform for dialogue and empathy.

## Introduction

This study was carried out as part of an overall research program investigating postvention (bereavement support) uptake and effectiveness in UK and Ireland adult males bereaved by suicide. The term “suicide loss survivor” (SLS) is utilized in this article as it is being increasingly used for those who have experienced the suicide of a close family member, or a friend, and face persistent, distressing trauma (1). It is thereby differentiated from survivors of suicide attempts. Against a backdrop of c. 760,000 annual suicide deaths globally (2), based on UK (3–5) and Ireland (6) government suicide statistics, annual suicides total approximately 7,500 with male suicide being three to four times more prevalent than female suicide. However, the UK is one of several countries where it has been suggested that the coronial system could result in a 15–50% underestimation of suicide as the officially determined cause of death. This has been associated with SLS pressure on the coroner, the coroner's inherent wish to protect SLS from the associated stigma and, most importantly, coronial reticence to declare deaths as suicide reflective of the legacy of deemed criminality (7). This may have been mitigated, more recently, by the standard of proof for suicide deaths, in England and Wales, being lowered to the civil standard of being on “the balance of probabilities,” from “beyond all reasonable doubt,” in 2018 (8). In the period 2000–2019, Ireland was in keeping with a global trend of declining suicide rates but the numbers for the UK increased, although not statistically significantly (2). The number of people bereaved by suicide annually, across the world, has been estimated at 48 million (9). Using an annual global suicide figure of 700,000 (noted in the same paper) the corresponding annual suicide exposure level, on a ratio basis, would be approximately 480,000 for the UK and Ireland. Using the estimate of 135 people being exposed to each suicide (10), the UK and Ireland annual exposure rate to suicide is 945,000. Results of a UK-wide survey indicated that 77% of participants (including professionals and those that lost friends) were majorly affected by losing a loved one to suicide, with half reporting mental health issues, 38% contemplating suicide, and 8% attempting suicide, directly related to their bereavement by suicide (11). Similarly, a survey conducted in Ireland found that 93% of SLS noted a moderate or major impact on their lives with mental health issues (56%), relationship struggles and alcohol abuse being typical factors, and 35% considering self-harm or suicide (12). A recent systematic review of the psychosocial impact of suicide bereavement on men highlighted their increased risk of suicide and mental health problems, plus issues with relationships and social interactions, over men not bereaved by suicide (13). Furthermore, a systematic review of postvention service models, between 2014 and 2019, found that, generally, studies of suicide bereavement support services had 80–91% female participation (14). However, evidence also suggests that there is no correlation between parental gender (or that of a child lost to suicide) and depression and grief (15). Similarly, no gender differences were found relating to siblings' suicide loss (16) or partner suicide loss, with both genders experiencing reduced life expectancy, higher rates of divorce, children in care, sick leave, and unemployment (17). Yet, despite encountering similar levels of grief and emotional distress to female SLS, male SLS seek help far less frequently. It has also been noted that the impact and management approaches of suicide bereavement interventions vary by gender, with the inadvertent emphasis on the effectiveness of postvention in largely female samples, thereby introducing a major gap in both postvention activities and the literature regarding men (18). In addition to SLS being prone to mental health issues and at increased risk of suicide themselves (19), understanding psychosocial influences, with appropriate interventions, has also been shown to be effective in lessening both suicidal behaviors and the likelihood of attempts (20). Therefore, men are typically not receiving much needed, proven, support to help them cope with their losses.

The purpose of this meta-ethnography was to identify qualitative research which summarizes current knowledge on how adult males experience, cope with, and adapt to, bereavement by suicide. Factors influencing this could be cultural, socioeconomic, educational, demographic, psychological, as well as previous history of trauma, which are implicated in postvention being available, taken up, and impacting its effectiveness. Based on a gained comprehension of adult male postvention research findings in the broad literature, the intent was to frame further novel research pertinent to the target population of UK and Ireland adult male SLS.

There are several approaches to qualitative evidence synthesis which have been collated, summarized, and “the state of the method” assessed (21). To achieve the intended objectives of this study, a method was required that goes beyond aggregation of themes but, rather, one which is interpretive of the underlying concepts facilitating creation of an overview regarding the fundamental issues faced by adult males bereaved by suicide. This reflects the two essential components of meta-ethnography, in viewing the source information as metaphorical, rather than literal, and translation of studies into each other during the synthesis (22). This approach allows for a derivation of commonality and the development of constructs that represent the dataset, as a whole, but may not individually be apparent in any of the studies included (23).

The goal was to assess prior research regarding how men have experienced, coped, or struggled with bereavement by suicide, along with any prevailing attitudes or perspectives reported. A secondary objective was to systematically review the level of male involvement in, and contributions to, relevant research. Finally, the intent was to consider whether the literature indicates male-specific factors relevant to the provision, and uptake, of postvention services.

## Method

### Search strategy

The search strategy involved retrieving records that describe the lived experience of adult males bereaved by suicide. The purpose of the search terms was to capture records covering suicidality, aspects of grief or bereavement, relationships to those lost, male involvement, and various types of qualitative study. Wildcards were used to capture linguistic variations, for example “grie^*^” allowing retrieval of records that describe grief or grieving. The search strategy was honed via multiple searches of the Academic Elite database. In conjunction with the research team and the [*University of West London*] specialist librarian, the following data sources were searched: Academic Search Elite, CINAHL Complete (Cumulated Index to Nursing and Allied Health Literature), Medline, PsycARTICLES, PsycINFO, ProQuest, and Google Scholar. These were selected as they are frequently used in psychological research and provide a broad coverage of relevant publishers. Each data source was searched independently, as they are indexed differently, and the search strategy was refined based on the initial output generated from the individual sources (see Supplementary material A for the complete search strategy).

### Search criteria

The searching of ProQuest was constrained by two of the primary terms, relating to suicidality and bereavement, plus limited to abstracts and the Google Scholar searches were of article titles only. Both measures were necessary to eliminate many thousands of extraneous hits and home in on the target records. The searches were set to return only English language records, to avoid interpretation bias, but no timeframe was set other than a publication date up to and including the end of November 2022^1^. There was no exclusion based on geography. All the studies included in this meta-ethnography had a significant level of male participation (50% or above) and similar aims, in terms of looking to elucidate how the experiences related to suicide-loss shape those left behind (see Supplementary material A for the complete search criteria).

### Approach

The overall approach adopted is compliant with that advocated by the “STARLITE” guidance which calls for the clear definition of the “**s**ampling strategy, **t**ype of study, **a**pproaches, **r**ange of years, **l**imits, **i**nclusion and exclusions, **t**erms used, **e**lectronic sources,” reflecting the mnemonic and intended to provide a standard for reporting literature searches (24).

To mitigate any inherent bias, in their consideration, the studies included in this meta-ethnography have been presented alphabetically by author [save for Ross et al. (25), which is embedded under Entilli et al. (26), as it is an earlier report of the same study]. The comparison of papers was then initiated by using the first study with each of the following studies being assessed against all those preceding (27). The analysis was conducted by Author #1 and cross-validated by Authors #2 and #3. All authors agreed that the themes and interpretative framework were embedded in the data and provided a meaningful interpretation of SLS's experiences^2^.

Studies were included that offered a valuable contribution to the synthesis. This is a subjective appraisal but is in keeping with meta-ethnographic practice (28). When conducting a meta-ethnography, it is common for the quality of the included studies not to be a basis for exclusion prior to the synthesis (27) and this was the case herein. There is no defined standard for assessing the quality of studies included in a meta-ethnography (29). For the purposes of this meta-ethnography, the CASP Qualitative Studies Checklist (30) was employed which consists of 10 questions. Application of these questions facilitated evaluation of studies based on key parameters, including methods, congruity of method with the aims and analysis, ethics, comprehensibility, and value in terms of adding to existing knowledge. Questions reflect three aspects of the quality review with items one to six focused on validity, seven to nine on the results, and 10 on the study value. This framework has been effectively used in other meta-ethnographies (27) where each study is assessed against nine questions, for specific aspects of quality, with ratings of “yes,” “can't tell,” or “no,” plus observations on the value of the research undertaken and overall comments. The studies were all assessed by Author #1 with the other two members of the team each reviewing a random sample. Any divergence of opinion was resolved through dialogue.

Meta-ethnography is a method for synthesis of purely qualitative studies, but it allows for a range of study designs to be included. Its systematic approach facilitates generation of insights into experiences and derivation of impressions and models that can inform both future research and real-world policy and practice (31). While meta-ethnography is an intricate method, and time-consuming, it is well suited for application to primary studies that contain rich data and are highly interpretative (31).

### Transparency and openness

This meta-ethnography adhered to the PRISMA 2020 guidelines for systematic reviews (32). All data and research materials (including our manual coding scheme) are available upon request. This review was not preregistered. Historically, meta-ethnographies have often lacked an audit trail, but standards, such as the eMERGe reporting guidance (33) employed herein, providing structure and aimed to enhance quality, robustness, and reliability, address that potential limitation (21).

### Research team

The full research team (of three) was involved at key stages (search strategy, extraction, translation, and synthesis) as well as the supporting researchers conducting randomly assigned, independent reviews where appropriate (10% studies post de-duplication, 10% full text review, and 50% quality assessment). Any differences in opinion, arising from such checkpoints, were collated for discussion at face-to-face team meetings and consensus reached prior to each stage being finalized.

### Positionality and reflexivity

The lead researcher is an SLS and is a trustee/volunteer with a UK peer-support group [*Survivors of Bereavement by Suicide*]. It is with that lens that the research described herein has been conducted and with an acknowledgment of the consequential potential for inherent bias. That said, the charity work has facilitated the lead researcher interacting with many other SLS who have lost a cross-section of loved ones, who come from a broad range of backgrounds, and their losses span from the very recent to 10 or more years ago. This has provided an expansive perspective beyond personal experience. Other members of the research team have not been bereaved by suicide and so brought a balanced viewpoint and provided a check regarding any intrinsic bias of the lead researcher, regarding the findings of this meta-ethnography.

As the primary executor of this meta-ethnography, the lead researcher inevitably perceived the development of the proposed model in the content of both personal experience and the interplay with a broad cross-section of other SLS. This was mitigated by the extensive experience of the overall team, with various qualitative research approaches, and the rigorous methodological approach employed herein to bring structure and transparency. The make-up of the research team facilitated the combination of academic rigor and real-life awareness of the impacts of suicide bereavement to inform the interpretation of the analysis of the studies included in this meta-ethnography. Thereby producing a novel and informative model that holistically depicts suicide loss survivorship and its overarching, life-changing complexity.

### Ethics

British Psychological Society ethical considerations were adhered to, including obtaining ethical approval from the [*University Research and Ethics Committee (UREC) at the University of West London*].

## Findings

The initial search yielded 1,645 records. After de-duplication, there were 1,189 records remaining. After the exclusion of gray literature, systematic reviews, quantitative studies, clinical trials, viewpoints, non-scientific records or those relating to fiction, studies lacking a focus on suicide bereavement, and studies with minimal adult male involvement, or where the findings are not attributable to the adult men that participated, a further 1,090 records were eliminated, leaving 99 to be sought for full text retrieval. During the review of articles obtained via the database searches (including Google Scholar) a number of papers were manually identified or cited in studies that were candidates for retention. In total these numbered 36, making the overall total to be sought for retrieval at 135. From both the database and manual search routes, a few papers were unavailable from the university library, therefore Inter-Library Loan requests were made. All but one (from the manual search path) were obtained, such that 134 records were retrievable overall.

**Figure 1:**
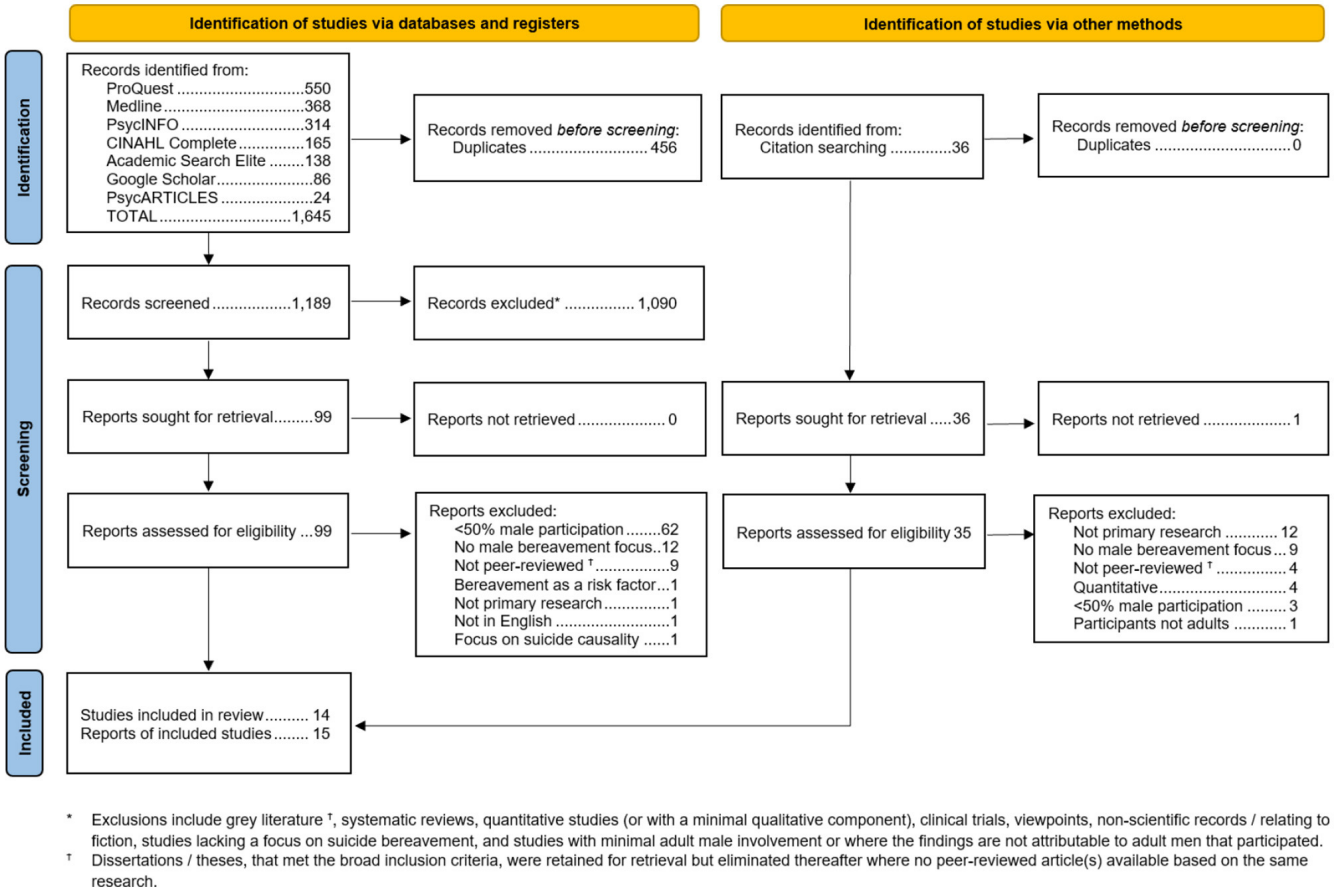
PRISMA flow diagram depicting the overall steps for identifying reports for inclusion in the meta-ethnography.

Figure 1 depicts the full screening process in line with the current PRISMA (Preferred Reporting Items for Systematic reviews and Meta-Analyses) guideline (32). It is based on the 2020 version of the PRISMA Flow Diagram template. One report retrieved (26) describes a longitudinal study of parents bereaved by suicide assessed at 6, 12 and 24 months after their losses. A prior report (25) is based on the same study but only covered the 6- and 12-month assessments. Both studies met the inclusion criteria set for the review but, following the latest PRISMA guidance on how this should be reported on the flow diagram, this represents a single included study and increases the “Reports of included studies” by one (34). Overall, based on consensus reached by the review team, 13 papers were included in the meta-ethnography arising from the database searches and two from the manual searches.

The quality assessment of the 14 studies indicated that, despite minor issues, all of them can be relied upon as valid data sources^3^.

### Study characteristics

All the included studies were conducted since 2010, with most (10/14) being from 2017 onwards. A summary of the key characteristics, from each included study, is presented in Table 1. Together, the two articles mapping to a single study are one of three Australian papers (#6 plus #7, and #1). Other geographies represented are Africa (1: Nigeria (#13)), Asia (4: China (#5), Hong Kong (#4), South Korea (#11), and Pakistan (#2)), Europe (4: England (#12), Germany (#3), Ireland (#9), and Poland (#15)) and North America (2: Canada (#8 and #14)). Cultures vary across these regions and/or sub-populations within them and, of the countries represented, suicide remains illegal in two countries (Nigeria and Pakistan). Eight of the 14 studies (#1, #2, #6 plus #7, #9, #12, #13, and #15) had both male and female participants while the remainder (#3, #4, #5, #8, #10, #11, and #14) included only men, with four of them based on a single participant (#3, #5, #10, and #11). The ages of participants were not supplied for three of the studies (#2, #5, and #12). Where ages were provided, many (6/14) included middle-aged to older participants (#3, #6 plus #7, #8, #11) with some (#4, #9, #13, #14, and #15) including a broad range of ages (4/14), and only two having exclusively younger participation (#1, and #10). Socio-economic background was not provided in most studies, but some did include specific information on the participant(s). In one (#10), it was noted that the individual was part of a family that had immigrated from a non-English speaking country and that they had experienced domestic violence, with a backdrop of financial difficulties. In the Nigerian study (#13) it was recorded that population, from which the sample was drawn, had strong literacy and religiosity, with employment primarily across agriculture, commerce, and the civil service. In the study conducted in China (#5), it was recorded that the participant was recruited from a bereavement support group in a city which is economically developed.

**Table 1:** Key characteristics of the studies included in the meta-ethnography.

**Paper**	**Country**	**Participant (s)^†^**	**Data collection**	**Method**	**Focus**	Aim(s)/Research question(s)^**^
1. Adams et al., (101)	Australia	Male = 4 (57%) Aged 20–27	Semi-structured interviews carried out in-person, via Skype or phone (breakdown not specified)	Interpretative phenomenological analysis	Young people bereaved by the suicide of a sibling	To explore the key issues in the **grief experiences of young adults bereaved by the youth suicide of a sibling**
2. Ali and Rehna, (102)	Pakistan	Male = 3 (50%) Age not stated	Semi-structured interviews carried out in-person at the participants' residences	Interpretative phenomenological analysis	Three parental couples bereaved by the suicide of a child	To explore **grief reactions and suicide bereavement in the context of stigma among parents**
3. Briggs et al., (103)	Germany	Male = 1 (100%) Aged 62 (treatment described for 7 years thereafter)	Case study of an outpatient attending a clinic at a university hospital	Psychodynamic psychotherapy	Psychotherapy of a suicidal man, who had suffered many losses including that of his mother to suicide	*The aim of this paper is to explore the **relationship between trauma and suicidal thoughts and behavior*** (Case study)
4. Chan and Cheung, (104)	Hong Kong	Men = 10 (100%) Aged 30–60	Semi-structured interviews carried out in Cantonese; location(s) not specified	Thematic analysis	Chinese men who had lost their wife or child to suicide	*This study investigated how **Chinese men*** **(bereaved by suicide)** ***communicated their sorrow and sought help***
5. Chen and Laitila, (105)	China	Male = 1 (100%) Age not stated	Semi-structured interview carried out in-person in a quiet, private venue	Assimilation model and the Assimilation of Problematic Experiences Scale (APES)	Widowed man recruited via a suicide bereavement support group in a developed city	*This study aimed to shed light on the **initial-stage bereavement experiences of an individual bereaved by suicide**, at 3 months from the loss of his spouse to suicide* (Case study)
6. Entilli et al., (26)^*^	Australia	6 and 12 months: Male = 7 (50%) Aged 50–68 (men) 24 months: Male = 6 (55%) Aged 56–68 (men)	Semi-structured interviews carried out in-person or by phone (breakdown not specified) at 6, 12 and 24 months after loss	Thematic analysis	Individual parents (no couples) that had lost a child to suicide	*The* [initial] *study* (25) *aims to examine the **individual experiences of both mothers and fathers bereaved by suicide over time**, specifically at the 6 **month and 12-month** time points after the death of their child./The* [follow-up] *study* (26) *aimed to extend the analysis over **24 months**, outlining the key themes of parents' suicide bereavement experience*
7. ^*^Ross et al., (25)	Australia	Male = 7 (50%) Aged 50–68 (men)	Semi-structured interviews carried out in-person or by phone (breakdown not specified) at 6 and 12 months after loss	Thematic analysis	Individual parents (no couples) that had lost a child to suicide	See above row
8. Ferlatte et al., (106)	Canada	Male = 2 (100%) Aged 40s	Photovoice interviews—location(s) not specified	Thematic analysis with hybrid inductive/deductive coding	Two men recruited form a local gay men's health organization	*This article presents a qualitative case study of two **gay men who lost a partner to suicide and explores how stigma may shape gay men's bereavement experiences***
9. Gaffney and Hannigan, (107)	Ireland	Male = 5 (50%) Aged 18−60+	Semi-structured qualitative questionnaire–location(s) not specified for face-to-face completion	Descriptive and interpretative thematic analysis	Members of two local bereavement support groups who had lost a partner or close family member to suicide	*This study aims to explore the **processes which enable SLSs to manage and cope during their tragic loss***
10. Jackson et al., (108)	Australia	Male = 1 (100%) Aged early 20s (11 years after the loss)	Face-to-face interview—location(s) not specified	Narrative case study	Case study of a man from a minority, migrant family who lost his uncle to suicide, when he was aged 13	*This article provides an important **first-person retrospective account of suicide survivorship through the eyes of a child***
11. Lee, Eunjin et al., (109)	South Korea	Male = 1 (100%) Aged 40s	Semi-structured interview—location(s) not specified	Interpretative phenomenological analysis	A man who lost his wife and only son via a parent-child collective suicide	*This case study explores one individual's personal experience as an **adult survivor of suicide who lost his wife and his only son through parent–child collective suicide** in South Korea*
12. Nelson et al., (110)	England	Male = 6 (67%) Age not stated	Semi-structured face-to-face interviews—locations agreed but not specified	Thematic analysis	Self-selecting, experienced ambulance staff from one service—all personally bereaved by suicide, including colleagues (not a requirement)	*The aim of this exploratory study was to **illuminate the experiences of ambulance staff in relation to attendance at a suicide**, including interaction with families bereaved by suicide at the place of death*
13. Ohayi, (111)	Nigeria	Male = 24 (89%) Aged 22–55	Semi-structured face-to-face discussions with a University Teaching Hospital pathologist, at their office, and the groups of relatives, both before and after the autopsies	Thematic analysis	Groups of relatives and non-relatives with connections to the community (2–5) for each of eight suicide (suspected) deaths	*The aim of this paper is to **highlight the perception and attitude of SLSs to suicide** in our environment*
14. Oliffe et al., (112)	Canada	Men = 20 (100%) Aged 20–63	Photovoice interviews—location convenient but not specified	Grounded theory	Adult men bereaved by suicide of a male friend, partner or family member	*What are the **connections between masculinities and suicide bereavement among men who have lost a male to suicide**?*
15. Ziółkowska and Galasiński, (113)	Poland	Male = 5 (50%) Aged 20–50	Semi-structure interviews–location(s) not specified	Discourse analysis	Adults bereaved by the loss of their father to suicide at least 2 years prior	*Taking a constructionist view of discourse, we aim to analyse **sons' and daughters' narratives in the context of two conflicting discourses of (positive) fatherhood and (negative) suicide***

^*^Ross et al. (25) is a report of an included study (PRISMA) with respect to Entilli et al. (26).

^**^Italicized text is verbatim, otherwise gleaned from the title, abstract and/or text.

^†^ Unless specified, the age range applies to all participants.

Most studies (#1, #2, #4, #5, #6 plus #7, #9, #11, #12, #13, and #15) collected data via semi-structured interviews (10/14). Data collection for one (#9) was unclear but an examination of the larger study of which it is a component (but not part of this meta-ethnography) implies that this was also via semi-structured interviews (35). Two studies (#8 and #14) employed photovoice interviews, and one was based on the psychotherapy notes for an individual (#3). The procedure, for the psychotherapy study (#3), also notes that the verbatim transcript was translated into English but no issues with this were mentioned. The authors of the study conducted in Poland (#15) noted that the participant quotes were translated from Polish into English and that some fidelity may have been lost due to the resulting text losing a degree of coherence, in some instances.

In terms of data analysis, Thematic Analysis (TA) was used in four of the studies (#4, #6 plus #7, #12, and #13), another used a hybrid of deductive and inductive TA (#8), and another noted the use of “descriptive and interpretative thematic analysis” (#9). Interpretive Phenomenological Analysis was employed in three studies (#1, #2, and #11). The remaining five studies each approached the analysis differently, one being based on a case study of psychodynamic psychotherapy (#3), with others employing grounded theory (#14) and discourse analysis (#15). Uniquely, in this set, one study (#5) applied ratings from the Assimilation of Problematic Experiences Scale (APES) to textual passages derived from the transcript. One paper describes a narrative case study (#9) but did not define an analysis approach, nor did it articulate any themes. It does, though, contain a rich set of participant quotes from which concepts can be gleaned.

### Analysis

The outcome of the translation process, involving iterative review of all the studies to assess for concepts consistent across them (reciprocal) or those that differ or are conflicting (refutational) and the subsequent interpretation to create novel themes (line of argument), is encapsulated in Table 2, in which, after a synopsis of each individual study, the cumulative concepts are listed. This reflects the research program's aim of understanding the broad impacts of suicide bereavement. Importantly, this table is not merely a list of themes, but attempts to portray the underlying, cohesive concepts arising from each study, and an amalgamation thereof.

**Table 2:** Main themes of the studies and cumulative concepts derived.

**No**.	**Paper/Country**	**Participant(s)^†^/ Type of loss/ Timeframe of loss**	**Main themes reported in study^§^**
1.	Adams et al., (101)/Australia	Male = 4 (57%) Aged 20–27/ Sibling/ Av. 3.75 yrs.	For themes with sub-themes: • The process of grief—initially, over time • Grief interactions—family, others and rituals • Continuing bonds—pre- and post-death • Meaning making and growth through grief
Concepts identified:			
• Trauma (shock, anger, fear, guilt, agony, grief, anxiety, denial, depression) • Questioning of own existence and purpose (including suicidal ideation) • Searching for answers and meaning/rationalization • Sense of abandonment • Shift in family/friend dynamics: harmony vs. divisive • Isolation, withdrawal, and avoidance • Stigma: judgement, disrespect, low empathy • Memorialization: lost futures, pride and personal growth, spirituality • Help and support: need/positive vs. negative • Protective toward other SLSs			
2.	Ali and Rehna, (102)/Pakistan	Male = 3 (50%) Age not stated/ Child/2–7 yrs.	For themes with sub-themes: • The psychological impact of grief—anger, agony, loneliness, grief, distress, helplessness • Stigma—gaze of others • Shame, guilt, and responsibility • Religion
Additional/updated concepts (M = **Modified**, N = New):			
• M–Stigma: judgement, disrespect, low empathy, **religious dogma** • N–Blame of self and/or others • N–Inner conflicts, e.g., in emotional pain but fear of losing the connection if it fades • N–Powerlessness (helplessness): diminished by the loss			
3.	Briggs et al., (103)/Germany	Male = 1 (100%) Aged 62/ Mother/32 yrs.	Three configurations: • The impact of suicide loss • Suicidality trauma • The impact of childhood trauma on suicidality
Additional/updated concepts (M = **Modified**, N = New):			
• N–Deceased's mental health/self-harm or suicide attempts • N–Childhood trauma, and expectations, shaping perceptions of suicide and suicidality			
4.	Chan and Cheung, (104)/Hong Kong	Men = 10 (100%) Aged 30–60/ Wife or child/ < 2 yrs.	Three themes: • Making sense of hidden grief • Processing guilt in bereavement • Stigma of masculine grieving
Additional/updated concepts (M = **Modified**, N = New):			
• M–Protective toward other SLSs (**masculine ideals**) • M–Powerlessness (helplessness): diminished by the loss and/or **loss of control** • N–Cultural taboo regarding suicide and mourning			
5.	Chen and Laitila, (105)/China	Male = 1 (100%) Age not stated/ Wife/ 3 months	• Community of voices—rationalizing, self-observation, self-regulation, normalcy of life • Problematic voices—uncontrollable emotions, accidental death, suicidal death. • Conflicting voices—Self-regulation voice vs. uncontrollable emotions • Normalcy of life vs. accidental death/suicidal death
Additional/updated concepts (M = **Modified**, N = New):			
• N–Focus on day-to-day routine/”normal life”			
6. 7.	Entilli et al., (26)^*^/Australia ^*^ Ross et al., (25)	Male = 7/6 (50/55%) Aged 56/50 – 68 (men) Child/ 6 and 12-month interviews	Three themes—interpreted sub-themes not supplied by the authors: • Searching for answers and sense-making—looking back, reaction • Coping strategies and support—avoidance, health, adaptation, support • Finding meaning and purpose—growth, harmony Same three themes in both papers—additional codes in the latter
Additional/updated concepts (M = **Modified**, N = New):			
• M–Searching for answers and meaning/rationalization, **frustration gaining information** • M–Shift in family/friend dynamics: harmony vs. divisive, **hyper-vigilance, fear of forgetting** • M–Isolation, withdrawal, and avoidance (**excessive working and/or drinking**) • N–Healthcare system: let down regarding the care of the deceased • N–Physical health problems			
8.	Ferlatte et al. (106)/Canada	Male = 2 (100%) Aged 40s/ Partner/ Not stated (>12 yrs. implied in one case)	Five themes: • Trying to prevent the inevitable • Guilty of keeping secrets • Dreams shattered by suicide • Abandoned and alone in grief • A lonesome road to recovery
Additional/updated concepts (M = **Modified**, N = New):			
• M–Trauma (shock, anger, fear, guilt, agony, grief, anxiety, denial, depression, **injustice**) • M–Deceased's mental health/self-harm or suicide attempts/**destructive behaviors** • M–Blame of self and/or others/**failure to keep the deceased safe** • N–Guilty of keeping secrets			
9.	Gaffney and Harrigan, (107)/Ireland	Male = 5 (50%) Aged 18–60+/ Partner or close family member/ 1–24 yrs.	Five themes across three phases (initial, medium- and long-term coping): • Initial experience of ‘shocked detachment' • Helpfulness of receiving support • Aiming to maintain a normal, familiar routine • Balancing emotional expression and regulation • Coming to terms with the reality of the loss
Additional/updated concepts (M = **Modified**, N = New):			
• M–Blame of self and/or others: failure to keep the deceased safe/**low self-esteem** • M–Deceased's mental health/self-harm or suicide attempts/destructive behaviors**/relief** • M–Protective toward other SLSs (masculine ideals)/**keeping strong for others**			
10.	Jackson et al., (108)/Australia	Male = 1 (100%) Aged early 20s (11 years after the loss)/ Uncle/ 11 yrs.	Three themes—interpreted sub-themes not supplied by the authors: • The pre-suicide period—tension, vigilance, stress and responsibility • Memories of the suicide—anticipation, responsibility, trauma • Post-suicide—withdrawal, isolation, guilt, hypervigilance
Additional/updated concepts (M = **Modified**, N = New):			
• N/A			
11.	Lee et al., (109)/South Korea	Male = 1 (100%) Aged 40s/ Wife and son/ Not stated	Two themes—interpreted themes not supplied by the authors: • Attributes of why the parent-child collective suicide occurred—maternal bond, culture • Complicated bereavement of heart-breaking losses—remorse, complicated grief, anger, trauma
Additional/updated interpreted themes and concepts (M = **Modified**, N = New):			
• N–Aspects of suicide and bereavement very specific to a culture			
12.	Nelson et al., (110)/England	Male = 6 (67%) Age not specified/ Public and colleagues/ Not stated	Three themes and sub-themes: • A profession under strain—lack of understanding of ambulance staff's role and low kudos, professional and personal bereavement • Responding to suicide in a professional capacity—impact, conflicting roles, dealing with the reactions • Lack of workplace support—lack of acknowledgment, reluctance to access work-based support, lack of training
Additional/updated concepts studies (M = **Modified**, N = New):			
• N–Professionals unprepared and unsupported in dealing with suicides and SLSs			
13.	Ohayi, (111)/Nigeria	Male = 24 (89%) Aged 22–55/ Relatives and community members/ Not stated	Along with an all-encompassing culture of denial, five themes—interpreted themes not supplied by the author: • Fear of stigma—ostracization, isolation, rejection of support • Fear of economic repercussions—hardship, superstition • Religion—family, fear of retribution • Shame—judgement, stigma • Anger
Additional/updated concepts studies (M = **Modified**, N = New):			
• N–Systemic broad and long-term denial • N–Economic hardship			
14.	Oliffe et al., (112)/Canada	Men = 20 (100%) Aged 20–63/ Male friend, partner, or family member/ Not stated	Two broad perspectives—interpreted themes not supplied by the authors): • Unforeseen suicide—perceived stoicism, shock, regret, meaning-making • Rationalized suicide—hypothesizing, powerlessness, reflection, retrospection, relief Additionally, across both perspectives—denial, detachment, protector, stoicism, societal expectations, emotions, awareness
Additional/updated concepts studies (M = **Modified**, N = New):			
• N/A			
15.	Ziółkowska and Galasiński, (113)/Poland	Male = 5 (50%) Aged 20–50/ Father/ 3.5–28 yrs. (mean 17.8 yrs.)	Conflict arising from two ambiguous sets of beliefs—strong fatherhood and the stigma of suicide: • The positive father– authority, “everything,” important • Distancing from suicide, rationalization, responsibility, agency
Additional/updated concepts studies (M = **Modified**, N = New):			
• N/A			
**Cumulative Interpreted Themes and Concepts from Studies 1–14**:			
• Trauma (shock, anger, fear, guilt, agony, grief, anxiety, denial, depression, injustice) • Questioning of own existence and purpose (including suicidal ideation) • Searching for answers and meaning/rationalization, frustration gaining information • Sense of abandonment • Shift in family/friend dynamics: harmony vs. divisive, hyper-vigilance • Isolation, withdrawal, and avoidance (excessive working and/or drinking) • Stigma: judgement, disrespect, low empathy, religious dogma • Memorialization: lost futures, pride and personal growth, spirituality • Help and support: need/positive vs. negative • Blame of self and/or others: failure to keep the deceased safe/low self-esteem • Inner conflicts, e.g., in emotional pain but fear of losing the connection if it fades • Protective toward other SLSs (masculine ideals)/keeping strong for others: • Powerlessness (helplessness): diminished by the loss and/or loss of control • Deceased's mental health/self-harm or suicide attempts/destructive behaviors/relief • Childhood trauma, and expectations, shaping perceptions of suicide and suicidality • Cultural taboo regarding suicide and mourning • Focus on day-to-day routine/”normal life” • Healthcare system: let down regarding the care of the deceased • Physical health problems • Guilty of keeping secrets • Aspects of suicide and bereavement very specific to a culture • Professionals unprepared and unsupported in dealing with suicides and SLSs • Systemic broad and long-term denial • Economic hardship			

^*^Ross et al. (25) is a report of an included study (PRISMA) with respect to Entilli et al. (26).

^†^Unless specified, the age range applies to all participants. § Abbreviated and/or paraphrased for space reasons.

When considering how studies are related to each other, originally characterized as metaphors to capture derived meaning across studies (28), more recently, such amalgamations have been described as storylines, which can help identify sub-sets, commonalities, and conflicts (22). No concepts, across the 15 papers, were found to refute each other. Concepts shared across papers were mostly reciprocal. A line of argument (28), or framework for interpretation was developed which is described below.

Following a method described elsewhere (27, 36), the 14 studies were tabulated to facilitate comparisons of the main concepts identified in each one (Table 3). For ease of review, only the main themes from each study have been included in the table and their descriptions abbreviated and/or paraphrased for fit. However, the full articles, including detailed analyses, findings and discussions, were examined during the translation process. With the proviso that, by their very nature, published studies included a selected set of quotations intended to represent the participants' experiences, such reflections in meta-ethnography are considered first-order constructs, and are typically located in the analysis or results sections of the articles. How participants' perceptions were interpreted by the study authors, are considered as second-order constructs, and usually described in the discussion sections. New concepts, synthesized from first and second order constructs, are deemed third-order constructs (37) and derived as part of the meta-ethnography analysis. From first, second and third order constructs, a line of argument can be generated: a framework of interpretation. Generation of a line of argument comprises an expanded comprehension of the underlying constructs to produce an extended or novel concept (22, 36). To illustrate the analysis, participants' quotations (first-order constructs) are presented in italics using quotations marks, whilst the authors' interpretations (second-order construct) are displayed using quotations marks only:

“*I got quite protective of Dad and Mum…but I think they were more worried about protecting me*.”/“Most participants reported that the death highlighted for them and their parents the fragility of life, triggering a pattern of reciprocal protection and anxiety for each other's safety” (Paper #1).

**Table 3:** Synthesis of concepts based on second- and third-order interpretations.

**First-order constructs (derived from participants)**	**Second-order constructs (derived from authors' analyses)**	**Third-order constructs (identified via synthesis)**
IMPACT: questioning of own existence and purpose (including suicidal ideation); searching for answers and meaning, frustration gaining information; powerlessness (helplessness): diminished by the loss and/or loss of control. “*What's the point?” Two important people in my life had been lost to suicide and I wanted to be with them.”* (101). “*The process of existence and mere survival is like a nightmare with no relief.”* (Ali and Rehna, (102)).	*Drinking excessively was still a recurrent behavior in males; however, some reported they had significantly reduced their alcohol consumption in order to improve their health* (26). …*men's emotions were about feeling and doing, drawing meaning rather than belaboring their bereavement and grief experiences around male suicide* (38).	**Changed Forever**: the impacts of suicide are not transitional but life-changing and permanent, even where SLSs are not locked into prolonged grief.
DISTRESS: shock, anger, fear, guilt, agony, grief, anxiety, denial, depression, injustice; childhood trauma, and expectations, shaping perceptions of suicide and suicidality; isolation. sense of abandonment; shift in family/friend dynamics: harmony vs. divisive. “*I felt exhausted and found it hard to talk to people about what had happened. I tried to stay away or get out of the house.”* (107). “*No one is phoning me. No one loves me. No one wants to talk to me.”* (106).	*Participants commonly described a number of emotional reactions of acute distress in the initial stages characterized most strongly by shock, pain, confusion, remorse, and guilt* (101). *A strong polarization among parents who were still oscillating in brooding rumination and those who have shifted toward sense-making could be observed* (26).	**Trauma**: the effect of bereavement by suicide is far reaching and the adaptation process non-linear; it can be exacerbated by other traumatic experiences and/or may be long-term.
BLAME: judgement, disrespect, low empathy, religious dogma. Self-blame leading to withdrawal, and avoidance (excessive working and/or drinking). “*In our religion suicide is prohibited.”* (103). “*…if anything, I've probably shut down a bit more to them. I've probably become a bit more insular.”* (25).	*The impact of suicide stigma was particularly apparent in Sheldon's and Nicholas's stories, first as they tried to connect their partners to support, and then after their partners' suicides when they sought support for themselves* (106). *The gaze of others establishes a rich and important contact signal, which we decipher by taking into consideration other characteristics of the appearance and also the contextual factors* (102).	**Stigmatization**: mental health issues, prior to or associated with suicide, are often frowned upon, and SLSs are frequently judged.
RESPONSIBILITY: failure to keep the deceased safe; low self-esteem; protective toward other SLSs (masculine ideals), hyper-vigilance/keeping strong for others. “*I remember after that situation my line of thinking at the time was so much disappointment in myself that I'd failed my uncle.”* (108). “…*had to keep strong for my kids as they had lost someone so special to them through suicide.”* (107).	*The bereaved men were reluctant to share their stories, as they wanted to avoid receiving criticism for having failed to prevent the suicide…* (104). *Most identified a growing closeness, particularly toward mothers* (101).	**Protector**: SLSs very often feel that they failed to protect those they lost; they frequently also feel responsible for subsequently keeping others safe from suicide.
MEMORIALIZATION: lost futures, pride and personal growth, spirituality; relief. “*I am alone. I have no dreams. My dreams are shattered. They are lost. There is nothing. It's empty.”* (106). “*I don't know what you call it, but I've got a book and I write, and I talk to him quite often in the book.”* (26).	…*described themselves as the casualties of their partners' deaths, with dreams of a future together shattered by the suicide*. (106). *The participants who had lost a child reported a high degree of fatherly pride* (104).	**Lost futures**: the lives that could have been are mourned, even as SLSs fondly recall those they lost; some take up activities their loved ones would have enjoyed and/or seek spiritual help; there may be relief post-bereavement where suffering has been ended.
HEALTHCARE and SUPPORT: deceased's mental health/self-harm or suicide attempts/destructive behaviors; healthcare system: let down regarding the care of the deceased; physical health problems; help and support: need/positive vs. negative; focus on day-to-day routine/‘normal life'; professionals unprepared and unsupported in dealing with suicides and SLSs. “*We all feel that the medical people let us all down, in that they didn't help John.”* (26). “*Whereas before, we'd try not drink for three days…but now it's definitely, at least one bottle to myself, every night.”* (25).	*The lack of trust in health professionals was explicitly displayed by some parents by a reduction in help-seeking attitudes or unwillingness to comply with medical treatments, either for a physical or a mental health related problem* (26). …*professional counseling services for gay men bereaved of a loved one by suicide need to be designed in ways that make them easily accessible, including promoting the services widely throughout the gay community* (106).	**Lost in plain sight**: many millions of people are significantly affected by suicide bereavement but are not recognized in terms of their losses, trauma and support needs.
CULTURE: taboo regarding suicide and mourning; aspects of suicide and bereavement very specific to a culture; systemic broad and long-term denial; economic hardship. “*Doctor, please don't say he died by suicide. We cannot allow the world hear that.”* (111) “*The suicide of my child is a shameful event for us as a family. As a father, I didn't dare to tell anyone about it because I would definitely be judged.”* (104)	…*migrant families often lack social and family support and so may struggle and become isolated when challenged by a family member with mental ill health* (108). *SLSs structure suicide narrative in a self-preservationist manner and therefore outrightly deny that the incident occurred* (111).	**Societal norms**: SLSs carry their experiences of suicide loss within the framework of their culture and society, often being constrained by established and acceptable customs which are not reflective of their needs.
INNER CONFLICTS: e.g., in emotional pain but fear of losing the connection if it fades; guilty of keeping secrets. “*After his death I spent a lot of time trying to imagine why he chose to kill himself. I was confused as I didn't see it coming.”* (38). “*We've got photos of him everywhere. But he's definitely disappearing.”* (26).	*Another aspect that made W's bereavement both complicated and distinctive was W's contradictory perception concerning his wife's death, veering between the two voices of “accidental death” and “suicidal death” (105). A dissonance seems to exist between participants' expectations of effective coping and the actual experience…* (107).	**Dualities**: SLSs of bereavement by suicide constantly face conflicting emotions, pressures, expectations, and needs with some causing cognitive dissonance.

**Figure 2:**
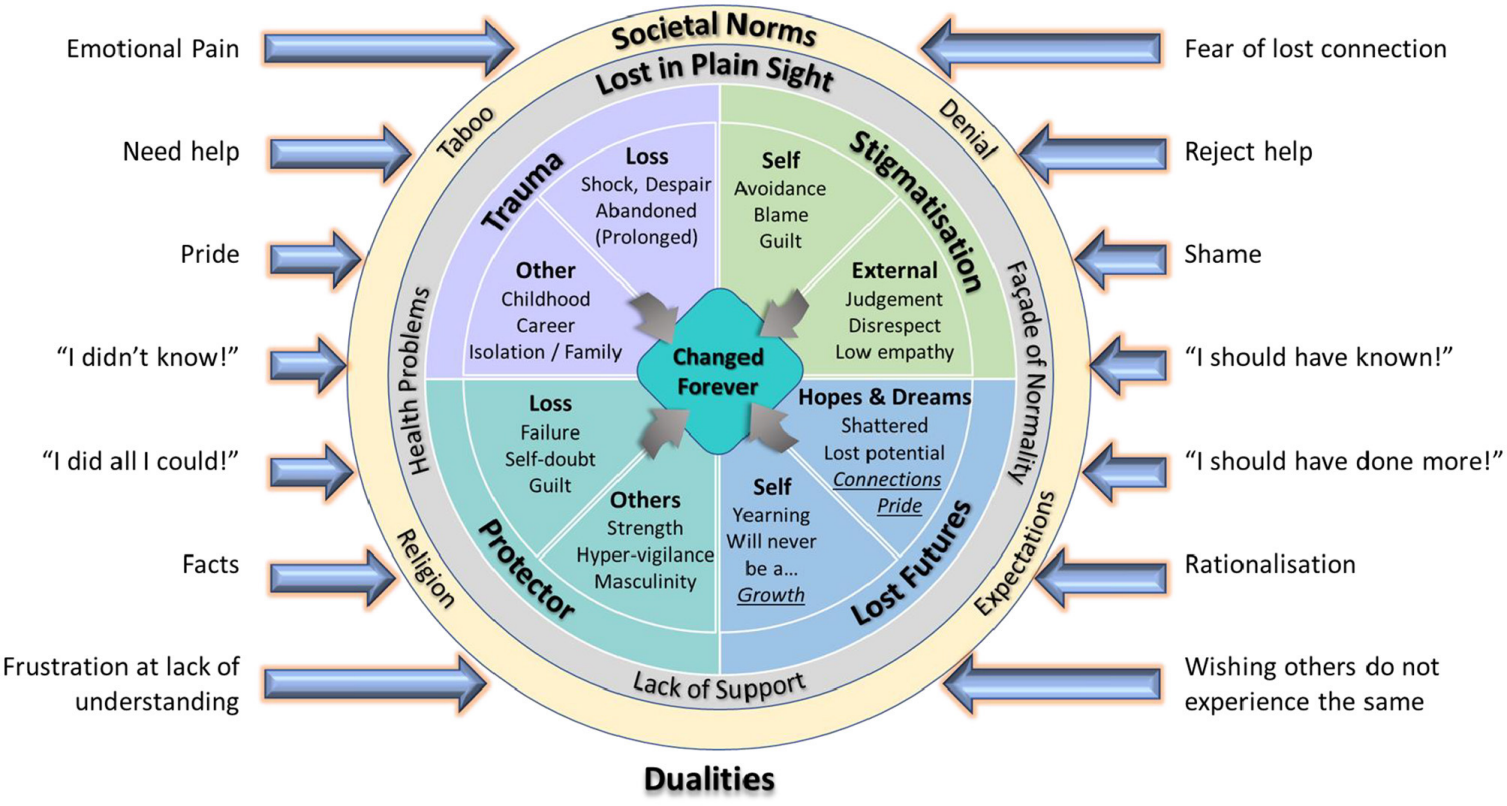
A proposed model of suicide bereavement impact on SLSs.

Table 3 reflects the reciprocal translations of the 14 contributing studies (15 papers). The first- and second-order constructs, included in the table, are representative and not exhaustive. The analysis identified eight concepts (third-order constructs), derived from eight themes (second-order constructs) which were used to generate an all-encompassing new “Suicide Bereavement Model” (line of argument). These concepts (themes) are: changed forever (impact), trauma (distress), stigmatization (blame), protector (responsibility), lost futures (memorialization), lost in plain sight (healthcare and support), societal norms (culture), and dualities (inner conflicts). The model demonstrates how these concepts interplay to reflect a shared core, inherent continuum of devastation mediated, and moderated, by external elements and competing drivers and factors such as relationships (to those lost and fellow SLS), culture, and timeframe (Figure 2). Other research has modeled aspects of the grief associated with suicide loss, from the perspectives of meaning-making and relationships to the deceased, the self and others (39) but the authors are not aware of any model that has holistically encompassed the multitude of factors associated with suicide bereavement.

### Changed forever

A universal experience is that SLS' lives are permanently and irrevocably changed (*Impact*): “*It's a thing I still constantly see when I'm asleep and I think of it every day while I'm awake...”*; first-order (Paper #6). There are many facets to this, including existential crisis, issues with trust, relationships, avoidance, withdrawal/isolation, and suicide risk. In keeping with the broader literature, from these studies it appears common for SLS to constantly struggle with trying to make sense of the suicide and derive some essence of meaning from their loss. They may be frustrated with a lack of hard information: “Some parents described their frustrations at trying to obtain information from coroners, psychologists and doctors in order to gain some understanding of the reasons for the suicide.”; second-order (Paper #7). Given the breadth, and gravity, of the upheaval and instability created by suicide bereavement, it seems reasonable to conclude that SLS are ‘**changed forever';** “*I was fragmented...broken that day when [Name] died. The process of existence and mere survival is like a nightmare with no relief.”;* first-order (Paper #2).

### Trauma

Another recurring thread was the intense emotional turmoil experienced by SLS, encompassing the immediate shock and horror but also angst and longer-term issues such as anxiety and depression (*Distress*); “Participants acknowledged initial feelings of depersonalization, detachment, blur, isolation/solitude, feeling robotic, and using strategies which created distance with the reality of the loss.”; second-order (Paper #8). Disbelief often propagates a sense of removal and detachment from reality such that SLS are overwhelmed and struggle to relate their level of devastation to those not in the same situation: “… participants in this study reported their feelings of comfort and relief in being understood and talking to others who had shared experiences.”; second-order (Paper #7).. ‘**Trauma**' associated with suicide bereavement can be long-term and potentially debilitating affecting not only individuals but whole families: “…*but Dad and I definitely got further apart. I was resentful toward him and I didn't understand his process [of grieving], it made me really angry*.”; first-order (Paper #1).

### Stigmatization

Often SLS feel judged by others (*Blame*) and treated with a lack of empathy: “*After the death of my son, I felt ignored by my family. They simply ignored my mourning. They always offer me unhelpful advice like it's time to move on, no one even tried to ask me how I was feeling from inside*.”; first-order (Paper #2). Being considered somehow liable for the factors that led to the death of their loved ones amplifies the trauma and distress with which they were already burdened: “…a mother reported feeling her living child directly blamed her for the suicide of her brother.”; second-order (Paper #6). There are also instances where those bereaved hold others liable for the death of their loved ones, within the family or outside of it, and were hostile toward them: “*I suppose I'll always question why the medical system had to let her down. I'm looking for somebody to blame, somebody's ass to kick. How did this happen? What could you do to prevent it?”;* first-order (Paper #7). Thus, ‘**stigmatization**' remains a pervasive issue that those bereaved by suicide frequently encounter: “*Will people stop asking questions? And if people do not ask questions now then what kind of stories will they fabricate?*”; first-order (Paper #2).

### Protector

Related to suicide stigma, it also appears common for SLS to hold themselves, at least in part, accountable for the suicide of their loved ones (*Responsibility*) as they considered it a failure to safeguard them: “*As much as people tell you that it's not your fault, it's impossible to not feel wrong for not being able to protect someone you love*.”; first-order (Paper #8). This is one area where some aspects of stereotypical masculine expectations were observed but was also noted as a factor for some female SLS: “Another example of avoidance was shown in fathers who reported working excessively in order to avoid the pain of thinking about their loss.”; second-order (Paper #7). The sense that they let down those they lost often leads to low self-esteem and low mood. At the same time, SLS often see themselves as being responsible for the safety and wellbeing of others impacted by the same bereavement: “Even as an adolescent, as the lone male in his immediate family, Joseph felt a responsibility to protect his female family members”; second-order (Paper #10). This includes being stoic to shelter others from their own emotions, assuming responsibility for the emotional care of others impacted by the suicide, and/or extends to hypervigilance due to an innate fear that others close to them may also be at risk of suicide. Both prior to the suicide they experienced, and thereafter, SLS frequently perceive themselves as being in the role of ‘**protector**': “They redefined their responsibilities to protect the rest of the family and educate others about mental disorders.”; second-order (Paper #4).

### Lost futures

The way in which SLS recall those they lost to suicide and commemorate them (*Memorialization*) seems multi-faceted. Some are left with a sense of emptiness and that their hopes and dreams have been crushed. Some embrace spirituality whilst others have their faith challenged. The latter may relate to the loss but also the way in which their faith, and other followers, view and treat suicide. In some instances, SLS embrace the hopes and wishes of those lost and attempt to emulate them and their goals. A positive aspect is that SLS can maintain, or enhance, strong connections, both to those they lost and other surviving friends and family, and often become driven to help others: “*I feel a lot better myself, it helps me by helping other people… I have finally got to a place where I can channel it into something constructive.”*; first-order (Paper #1). In one instance (Paper #14) a SLS noted a sense of relief, when they recollect their loved one, because the person lost to suicide was suffering and self-destructive. Some bereaved individuals recall their loved ones with pride, which may be psychologically protective. How SLS experience continuing bonds with their decedents varies depending upon the relationship to the person lost and their gender, the expectations of others, whether they had been able to come to terms with the loss, time since bereavement, and the gender of the bereaved. There are cases where, in addition to their bereavement, SLS mourn “**lost futures”** such as their decedents being present at a meaningful family event or that they will never be known to younger family members or those yet to be born: “*What could they be doing now? Could they have got married and had kids?*”; first-order (Paper #1).

### Lost in plain sight

Discontent with the provision of healthcare can pre-date bereavement, and was not uncommon, where those lost were seeking or were under treatment prior to their suicide. SLS relate that they considered that their decedents were let down and services were lacking or insufficient: “*I suppose I'll always question why the medical system had to let her down*.”; first-order (Paper #7). It was also noted that professionals are often unprepared, and unsupported, in dealing with suicides and SLS and, in some cases, SLS find the attitude and abilities of professionals inappropriate and inadequate. SLS also note a lack of transparency regarding the support available to them (*Healthcare and Support*). They can feel alienated by those within their social network actively avoiding them, but may also contribute to their isolation by maintaining a façade of normality, and feigning coping, whilst enduring inner turmoil: “*I'm afraid to talk to my friends because they will ask me about my children, so I avoid them. If we do see each other, I avoid the topic by changing the subject*.”; first-order (Paper #4). Whether externally or internally imposed, issues with obtaining professional support, and/or the distancing of SLS from people they interact with daily, can result in them being ‘**lost in plain sight**' as their plight goes largely unnoticed, or disregarded, by those around them: “*I did go to pieces when alone at night*.”; first-order (Paper #9).

### Societal norms

Beyond the broad stigma associated with suicide, SLS also may have to cope with taboos and systemic denial within the communities in which they live (*Culture*): “In terms of time of the suicide, the cultural factors present may influence how an individual reacts and copes in the aftermath, as stigma and cultural attitudes about suicide and grief seem to have a key role.”; second-order (Paper #2). Within suicide-bereaved families, there may be unspoken conventions of never discussing the event, and some people even attempt to hide the cause of death from others. This can create an atmosphere of secrecy, such that those not present or very young, at the time of the suicide, are never given a true account of events. Men can be especially prone to regarding suicide as a taboo subject. External to the immediate family, there may be taboos surrounding suicide that arise from religious beliefs or are specific to certain ethnicities: ”*In our religion suicide is prohibited. In Islamic law, it is forbidden. I believe that he has reached his destination but he is not in peace. We prepare ourselves for the real-life [life after death] but not this way.”*; first-order (Paper #2). In addition to the taboo of suicide, in some cases SLS face, or are party to, outright denial and secrecy regarding the manner in which their loved one died: “*Doctor, please don't say he died by suicide.... No, doctor, please, nobody must hear that. You can say anything but... please just don't say that.”;* first-order (Paper #13). Pressures on SLS, to withhold their losses, vary by culture and religiosity but, in all cases, the extent to which their bereavement is shared, acknowledged, and accepted falls within ‘**societal norms**'. A lack of certainty, regarding how their struggles are perceived, can exacerbate avoidant behaviors and contribute to their sense of the world, as they know it, being undermined: “While they felt that men should not seek help, they also felt that if suicide was not a taboo topic in their support group, they could more easily share their feelings within the group.”; second-order (Paper #4).

### Dualities

Collectively, the concepts indicate that SLS often experience opposing emotions or viewpoints simultaneously (*Inner Conflicts*), and this is exemplified by the study of those bereaved by the suicide of their fathers: “Founded by a conflict between two social metanarratives: stigmatizing suicide and strong fatherhood, we argue that it is underpinned by ambiguity.”; second-order (Paper #15). This can be extended, as part of the line of argument, that is characteristic of meta-ethnography, to cover other “**dualities”** faced by SLS. These include aspects such as SLS logically knowing there was nothing they could have done to prevent the suicide, but feeling guilty nonetheless, or feeling guilt that they let their loved one(s) down even though they know they did all they could for them prior to their deaths: “*…my line of thinking at the time was so much disappointment in myself that I'd failed my uncle. That was a burden that I carried with me just forever. Probably last year or this year was the first time in which I sort of now realized that maybe I shouldn't be so hard on myself.”*; first-order (Paper #10). One of the studies (Paper #5) describes vacillations between acceptance and denial of suicide plus the struggle to maintain an outward control while constantly dealing with “uncontrollable emotions.” SLS can also be stuck between wanting to mitigate their feelings of loss and being constantly reminded of their trauma: “*Whenever I thought of reducing the grief, I remembered my son, and more than that the way he died never get away from my eyes*.”; first-order (Paper #2). They can also perceive a disconnect between their own expectations of coping in contrast to the persistence of their grief (Paper #9). Overall, there seem to be a number of dualities, that those bereaved by suicide perceive, such that they often encounter conflicting notions concurrently.

### Summary—A novel model for suicide bereavement

The far-reaching, and long-standing, impacts of suicide bereavement with, at its core, the permanency of its life-changing implications, are the basis of the model (line of argument) constructed to describe, and contextualize, the synthesis of concepts gleaned from the 14 studies. This is encapsulated visually in Figure 2, and is proposed to be transferable, fitting suicide bereavement regardless of gender, demographics, relationship to the person lost, timeframe and culture. The proposed model is intended to represent the factors that influence adult SLS' cognitive and behavioral changes when experiencing bereavement by suicide, based on the 14 studies assessed. The deep-seated and fundamental modification to SLS' world view is manifestly impacted by key factors that pervade their lives. There is the emotional shock, often exacerbated by underlying issues and/or prior events, and entrenched grief, intensified by blame (externally and/or internally generated) which frequently results in stigmatization. While SLS struggle to cope with their losses, they may also constantly yearn for futures they can never attain with an associated sense of failure, regarding the safety of those lost, and foreboding, regarding others close to them, which can lead to a constant self-doubt of their capabilities to shield those they love. Surrounding all these very strong reactions, SLS may not get appropriate support, or acknowledgment of the impacts on their health, and feel constrained to outwardly project a level of normality, in their everyday lives, that masks their real, and deep-rooted, psychological state. This is driven by factors including culture, religion, and what they perceive to be expected of them by society, as a whole, or components thereof, such as family members, the workplace and community leaders. The combined pressure of all of these factors, bearing on them in unison, can result in SLS experiencing constantly competing perspectives regarding the circumstances of their bereavements.

The primary objective of this meta-ethnography was to investigate aspects of male suicide bereavement, whereas the model is not gender specific. This is because the analysis did not identify any characteristics that could be exclusively attributed to male participants and most of the studies also included female participants. The secondary objective of this review, to gain insight into how those factors impact SLS' inclination to seek or avoid support, and to continue or not continue with support involvement, is addressed to some extent by the model, but an apparent lack of evidence, from the 14 studies assessed, reinforces the need for further research to elucidate the barriers and enablers relevant to postvention uptake and continuance.

## Discussion

The aim of this meta-ethnography was to identify prior qualitative research which summarizes current knowledge on how adult males cope with, and adapt to, bereavement by suicide. Overall, the findings suggested that men coped by both extracting meaningfulness and protecting themselves by emotionally separating from the event. While these studies suggest recognized “masculine-style” of grieving, such as stoicism, fatalism, anger, and over-working, they did not get to the essence of what makes men deal with suicide differently from a “conventional-style” of grieving, typified by women, whereby grievers more openly share their feelings and, consequently, received greater support (40). The Suicide Bereavement Model, described herein, is proposed as being applicable to all adults due to the translation synthesis, core to the meta-ethnography, indicating that the included studies support a pertinent line of argument, from which the model is derived, regardless of gender. Of the papers where participation was limited to men (#3, #4, #5, #8, #10, #11, and #14) the focus was on individuals or narrowly targeted groups. Of the studies including males and females (with at least 50% male participation), very little was noted as specifically attributable to men, and the nature of thematic illumination lends itself to inclusion of participants quotes that best exemplify each theme, often masking whether, or at least not amplifying, any trends in gender alignment with the aspects under review. Overall, based on the information gleaned from the 14 studies that fell into the meta-ethnography, despite some instances of male survivors demonstrating stoicism and assuming the role of protector, it was considered that the syntheses and line of argument are equally applicable to men and woman, as some papers indicated the similar traits emerged in female participants. Therefore, the discussion, and associated Suicide Bereavement Model, is proposed to be applicable regardless of gender.

### Changed forever

The review findings reveal that suicide bereavement is complex and has many interwoven facets. These wax and wane, interact, and can have differing individual or collective impacts on SLS' capabilities to adapt and cope, in the immediate term after their loss, but also for many years to come, as they struggle to derive any meaning. In that, the overall impact of bereavement by suicide changes SLS' view of the world, and is interwoven into their belief system, attitudes, and daily behaviors, it can be regarded as a form of embodied biographical disruption (41). The complexity regarding SLS' drives and needs, with respect to making meaning out of their losses, has been noted elsewhere (42). That said, even as those bereaved by suicide process their losses and re-shape their lives, the evidence, herein and in the literature, suggests how they handle their grief is far from linear (43, 44). Rather grief intensity can fluctuate and be reactivated by both internal and external stimuli. While some SLS demonstrate personal growth, partly characterized by strengthening existing relationships and facilitating forging new ones (45), over time, some are often fundamentally lonely (46) and locked into negative patterns of “prolonged grief” (47)—now recognized as a mental health condition, evolving from what was previously described as “complicated grief” (48). Indeed, the combined shifts in SLS' perceptions, experiences, and personal reassessments of every aspect of their lives, are very common resulting in their lives being changed forever.

### Trauma

Following suicide bereavement, typically “acute grief” manifests as powerful and disturbing trauma. Psychological shock can be protective, in the short to mid-term, and prevent an individual from being overwhelmed with gloom and desolation. This can allow them to deal with the administrative aspects of death such as registrars, funeral directors, travel, and finances (49). While it has been suggested the difference between suicide bereavement and other types is negligible (50), others have argued that it is very much different, supporting that those bereaved by suicide experience psychological distress above and beyond that associated with other types of loss (19, 51). Men's expression of grief may be delayed, with respect to that of women (52). SLS can feel as if they also lost part of themselves when their loved ones took their own lives (53, 54). In keeping with some of the studies that form part of this meta-ethnography (Papers #3, #10, and #12), it has been observed that other traumas, in the lives of the those bereaved, can significantly impact how they deal with a loss to suicide (55). The trauma, related to suicide, is not limited to individuals but plays out within, and across, family and social constructs (56, 57). However, the majority of studies on suicide focus on specific relationships to those lost (58). Little is known regarding the overall dynamics and how suicide loss impacts generationally (59). Suicide-related trauma can be exacerbated by the bereaved feeling isolated individually (60) or as a family (61) and can be compounded by pre-existing trauma (62). Bereavement by suicide can lead some SLS to question their own existence and undermine their prior value systems (43, 63).

### Stigmatization

The stigma associated with suicide is widely recognized as an issue for SLS (64). Where decedents had known mental health issues and/or prior suicide attempts, stigmatization can be prevalent even before their losses (65). SLS sense that, generally, people do not perceive suicide as something that can happen in their families, and the families where it does occur are somehow blighted (66). The bereaved can be viewed as sullied by their loved one's suicide, with outsiders assuming mental illness pervades the family, and that it must be prone to suicidality (67). In some cultures, stigmatization of SLS can arise, and be very strong, within families and communities, as noted in a couple of the studies included is this meta-ethnography (Papers #2 and #13) and elsewhere (61, 68). The potential sequelae of suicide bereavement can further lead to stigmatization, as noted for sufferers of prolonged grief disorder (69). Not only are SLS subject to external stigma, but they may also internalize the unempathetic and judgmental opinions of others (70), thereby attributing to themselves blame, and subsequently shame; such self-stigmatization can lead to SLS adopting non-adaptive thinking and behavior (67). Perceived blame can intensify the negative connotations of suicide loss and magnify the suffering of SLS (71, 72). All facets of blame, in combination, can be exacerbated by inaccurate and inconsiderate media coverage (73).

### Protector

As noted in the findings of several of the 14 included studies, SLS often berate themselves for failing to protect their decedents (Papers #3, #4, #8 and #10). This has also been reported elsewhere (74). The assumption of the role as protector, to other SLS, was also noted (Papers #1, #4, #6, #9, #10, #12, and #13) and has been observed more broadly, including female SLS “directing from the shadows” (75) and SLS looking to rescue others in potential suicidal stress (76). With respect to parents that had lost a child, one paper (#6) reported that fathers assumed the role of protector, with respect to their partners, as they perceived the latter requiring support above and beyond their own needs. Elsewhere, men have also been noted to assume the role of carer, toward their female SLS partners and families (13). Where striving for such safeguarding results in SLS becoming hypervigilant, or overprotective, there is an increased emotional burden to bear, on top of individual's own grief (56).

### Lost futures

While SLS grieve intensely, related to the loss of a loved one to suicide, and its aftermath, they also speculate on what might have been in terms of their decedent's potential achievements, time and activities they could have shared, and the children they will never have (53, 54). These aspects can also cause an extension and prolongation to their grief (43). SLS often have a strong desire to keep the memories of those they have lost alive and close to them. While generally beneficial, this has been found to add distress where they could not maintain a cohesive bond, due to loss of mementos, waning memories, or a sense that they have lost control of how their relationship to their decedent is manifested (77). Increasingly, memorialization of those lost to suicide is via social media and online platforms (44). While this aspect of grief management is relatively new, there has been research suggesting mixed effects, with indications that SLS can both find an outlet for their emotions and maintain a virtual relationship with the loved one lost via dynamic media (78), but that commemorative internet sites can exacerbate anguish due to a resulting exacerbation of emotional stress (79).

### Lost in plain sight

Somewhat as a result of the persistent taboo around suicide, SLS tend to adopt a façade of normality hiding, or even denying, their true feelings (54) with men being more prone to, or even primed for, emotional concealment (80). Such a persona may be maintained in public, but the mask sometimes drops in the context of peer support (81). SLS can be overwhelmed and struggle to relate their level of devastation to those not in the same situation (82) and maintenance of a pretense of not needing help, or a lack of trust in the provider, may result in those bereaved lacking much needed support (83). Men are often unwilling to consult professionals for fear of being perceived as ineffectual and incapable (52). Also, due to suicide not being openly discussed, SLS may be unaware of its prevalence thereby missing the opportunity of support from others similarly afflicted (84). Men are generally assumed to be outwardly more stoic and are noted to use avoidance, such as a focus on work (85) or increased alcohol consumption (86), as part of their grieving pattern. This, though, can be the perpetuation of a gender stereotype and lead to them perceiving that their needs are being ignored (52). Overall, the lack of attention to SLS' needs can create a scenario where they live “under the radar” as they continue to be faced with significant turmoil, in relation to both themselves and those around them, encompassing their role as a protector, the potential futures lost to them by the suicide, stigmatization, and on-going trauma. SLS are prone a variety of mental health issues that have been found to vary by gender, with men being less likely to seek help (87) and their needs not being identified in healthcare policy setting (80). Research has also identified an increased risk of physical ailments in SLS (88) however, they may be less likely to engage with healthcare services if they perceive that those providers did not meet the needs of those lost to suicide (38, 89). In some cases, organizations can lack empathy (46) and be misaligned with the requirements of the bereaved, with healthcare professionals demonstrating a lack of thought and awareness (90) or frustrating SLS by not providing much needed information (91).

### Societal norms

The model depicting the multi-faceted negative experiences of SLS, presented herein, does strongly disagree with the notion of a linear nature to grief and bereavement, and other studies have also indicated that suicide bereavement is very much non-linear (92), despite SLS being pressurized, by external expectations to adhere to a broadly accepted sequence of stages exemplified by linear grief models (93). A recent systematic review of male suicide bereavement studies noted that men often experience marked grief responses but are constrained by the accepted customs and anticipated masculine behaviors in society (13). Several of the 14 included studies noted contextual social and cultural taboos, regarding openness and acknowledgment of suicide, with some highlighting deeply rooted negativity and denial (Papers #4 and #13). The level of repudiation seemed to reflect cultural hostility toward suicide, and SLS, and encompassed religiosity. Taboos can be faced at a family level, where members refuse to engage in a dialogue regarding a suicide (71), or can be at a cultural level, driven by the fear of repercussions and/or religious dogma (68, 94). The latter is exacerbated with suicide remaining an illegal act in many countries (95). Losing someone to suicide has been found to both enhance religious beliefs and diminish them, depending on the individual (96). Lack of empathy toward SLS, as a broad cohort, can lead to disenfranchised grief (44) and the judgement by others may be part of an overall stereotyping of bereaved families as being somehow broken or dysfunctional (61, 67). The need for better education, and a shift in societal attitudes, has been noted as necessary for SLS to receive both compassion and meaningful support (97).

### Dualities

Some of the papers assessed (Papers #2, #6, #8 and #9) suggested their findings exemplified the Dual Process Model of Bereavement's oscillation between factors associated with loss and those that help restore the bereaved to a level of normality (98). The model herein is not in conflict with it, as the “dualities” described here could reflect the “oscillations,” between “loss-oriented” and “restoration-oriented” behaviors, portrayed in the Dual Process Model. That said, the proposed model suggests that the experience may in fact be more intricate, in that SLS may not necessarily find themselves gravitating to either pole, at any given point in time, but may experience both concurrently. For example, they may intellectually accept that they did not have prior knowledge of their loved ones' struggles but, at the same time, still carry guilt because they cannot escape the feeling that they should have known. Whilst not wishing anyone else to experience the trauma of suicide bereavement, at the same time SLS frequently feel that they are not understood (81) or recognized (99). One of the included studies (#4) contains multiple examples of this type of conflicting emotions/experiences, such as emotional regulation vs. uncontrollable emotional responses, which fight for dominance. So, instead of merely oscillating between aspects of bereavement, the proposed model supports that SLS often experience both faces of the dualities at the same time, that is, they co-exist in perpetual contention. Elsewhere, such opposing drivers have been described as leaving SLS in a perpetual liminal state (100).

### Implications

The proposed Suicide Bereavement Model brings together perspectives across both the studies included in this meta-ethnography and the broader literature. It could be used in practice (clinical, therapy/counseling, education, peer support) to better understand the highly complex and interwoven components of suicide bereavement, thereby facilitating improved and extended services available to SLS that are more in-tune with their needs. A common perception, among SLS, is that those not bereaved by suicide can never understand their situation. While the model cannot confer full comprehension of suicide bereavement, it can go a long way to assist those looking to assist SLS by providing a platform for dialogue and empathy.

### Strengths, limitations, and reflexivity

The 14 primary studies are all relatively recent (2010 and after), demonstrating modern approaches, and adopted recognized qualitative research methods. Adopting the eMERGe project guidance (33) provided structure and rigor to the process of conducting this meta-ethnography. A simplistic (alphabetical by author) approach for synthesizing the studies was adopted to mitigate any potential bias regarding the study locations, demographics, methods, and findings. Throughout its development, the team focused on a clear rationale, distillation of appropriate insights and extrapolation of concepts, a pragmatic and critical assessment of findings, and the creation of a clear model, that describes the output, to achieve high quality at each process stage, and overall (29). There was a strong accord within the research team throughout the stages of the meta-ethnography and the derivation of the model that describes suicide bereavement holistically. The model proposed is novel, with its generally applicability bolstered by the inclusion of studies with a high proportion of male participation, and it seems to resonate with the broader literature on the impacts of suicide bereavement.

Assessment of the 14 studies highlighted some stereotypical male behaviors, such as stoicism and assumption of gender specific roles, but did not elucidate detailed gender variations in terms of accessing and embracing support. However, that emphasizes that this remains an under-researched area. Another potential limitation is that study authors selectively include quotes, from participants, to illustrate a theme, but there may be numerous other quotes, that were not included, from participants. It is possible that papers with lower male involvement (excluded from the present review) could contain information of relevance to male attitudes to suicide bereavement and support. As is the case with most psychology research, the majority of participants originated in ethnically European dominated locations, with limited cultural homogeneity, potentially limiting the transferability of the model more broadly. Overall, this meta-ethnography emphasized the dearth of research into postvention in general, and especially regarding its applicability to men, indicating the need for further research.

## Conclusion

The research team is not aware of any other model that comprehensively describes suicide bereavement. It portrays that suicide bereavement is highly complex with many interwoven facets that are constantly interacting and can be in contention. It is therefore important that those providing support services to SLS are aware of this complexity and recognize that, while addressing individual components of suicide bereavement, such as the initial grief and guilt, may make sense pragmatically, they need to be considered in the context of the totality of SLS' long-term needs, and not seen as isolated goals or endpoints. SLS strongly believe that suicide bereavement is different to other types of loss, which is supported by qualitative studies, and thus healthcare professionals, emergency service personnel and coroners should acknowledge this and act accordingly. Suicide bereavement permanently shifts SLS' perspectives and any improvement in coping, or personal regrowth, is not a linear process but cyclical in nature with the likelihood of many setbacks, often triggered by anniversaries, birthdays, holidays and other key dates or events, such as a family celebration from which the person lost is conspicuously absent. Those that deal with the suicide bereaved need appropriate training to be able to interact with them effectively, and with sensitivity and empathy, to meet their needs.

Most of the research into suicidology has a focus on prevention, which is highly important but does not cover the impact of suicide on those left behind. Research into the implications for men bereaved by suicide remains a rarity and understanding the needs of men plus, crucially, the reasons why many men do not engage with support services is an under-investigated aspect. The numbers of men that have historically participated in suicide research are low and those that do so may be atypical due to their willingness to share their experiences. Papers published where participants are mixed gender may mask factors especially pertinent to men as researchers chose quotes best suited, to make a given point, which may come from a female participant even though the issue in question may have been predominately a concern to the men in the study. More evidence is needed on why men mostly avoid suicide support/research and how they can be encouraged to participate. Some studies identify avoidance behaviors in male SLS, including over-working and excessive alcohol consumption, but these are symptomatic and do not get to the underlying motivators. Studies often reinforce male gender stereotypes which might perpetuate their grief being masked or even suppressed. Hence the Suicide Bereavement Model is being proposed as being equally applicable to men and women, as there is insufficient evidence to differentiate at this point in time.

## Data Availability

The data analyzed were from publicly available published academic papers.

## Footnotes

^1^The Chen and Laitila paper met the criteria at the time, as a pre-publication, but has since been formally published in 2024.

^2^Further methodological details are available from the corresponding author.

^3^Further quality assessment details are available from the corresponding author.
