# Supplementary material for: A suicide bereavement model: based on a meta-ethnography of the experiences of adult suicide loss survivors
Source: Front Public Health. 2025 Jul 14;13:1596961. doi: 10.3389/fpubh.2025.1596961 (PMC12301309; doi:10.3389/fpubh.2025.1596961)
Supplement: Supplementary file 1 [file Supplementary_file_1.docx]

Supplemental Material A – Search Strategy & Criteria

| Database / source | Search terms |
| --- | --- |
| Academic Search Elite | (suicid* OR self-killing) AND (grie* OR bereave* OR mourn* OR loss) AND (survivor* OR relative* OR relation* OR dependant* OR family OR parent OR spouse* OR widower OR child* OR sibling* OR orphan* OR friend*) AND (male* OR men OR man) AND (qualitative OR interview OR ethnographic OR phenomenological OR experience*) |
|  | Apply related words 🗸 \| Apply equivalent subjects 🗸 \| Language = English |
| CINAHL Complete | (suicid* OR self-killing) AND (grie* OR bereave* OR mourn* OR loss) AND (survivor* OR relative* OR relation* OR dependant* OR family OR parent OR spouse* OR widower OR child* OR sibling* OR orphan* OR friend*) AND (male* OR men OR man) AND (qualitative OR interview OR ethnographic OR phenomenological OR experience*) |
|  | Apply related words 🗸 \| Apply equivalent subjects 🗸 |
|  | Language = English \| Limits: Human \| Sex = Male \| Age Groups = All Adult |
| Medline (OVID) | (suicid* OR self-killing) AND (grie* OR bereave* OR mourn* OR loss) AND (survivor* OR relative* OR relation* OR dependant* OR family OR parent OR spouse* OR widower OR child* OR sibling* OR orphan* OR friend*) AND (male* OR men OR man) AND (qualitative OR interview OR ethnographic OR phenomenological OR experience*) |
|  | Limits: Humans \| English Language \| Male \|  Age Groups = All Adult (19 plus years) \| Map term to sub-heading 🗸 |
| PsycARTICLES | (suicid* OR self-killing) AND (grie* OR bereave* OR mourn* OR loss) AND (survivor* OR relative* OR relation* OR dependant* OR family OR parent OR spouse* OR widower OR child* OR sibling* OR orphan* OR friend*) AND (male* OR men OR man) AND (qualitative OR interview OR ethnographic OR phenomenological OR experience*) |
|  | Apply related words 🗸 \| Apply equivalent subjects 🗸 |
|  | Age Groups = Adulthood (18 years & older) \| Population Group = Male \| Language = English (post query) |
| PsycINFO | (suicid* OR self-killing) AND (grie* OR bereave* OR mourn* OR loss) AND (survivor* OR relative* OR relation* OR dependant* OR family OR parent OR spouse* OR widower OR child* OR sibling* OR orphan* OR friend*) AND (male* OR men OR man) AND (qualitative OR interview OR ethnographic OR phenomenological OR experience*) |
|  | Apply related words 🗸 \| Apply equivalent subjects 🗸 |
|  | Age Groups = Adulthood (18 years & older) \| Language = English \| Population Group = Male |
| ProQuest | (suicid* OR self-killing) AND (grie* OR bereave* OR mourn* OR loss) [in ABSTRACT] AND (survivor* OR relative* OR relation* OR dependant* OR family OR parent OR spouse* OR widower OR child* OR sibling* OR orphan* OR friend*) AND (male* OR men OR man) AND (qualitative OR interview OR ethnographic OR phenomenological OR experience*) [in ANYWHERE] |
|  | Language = English |
| Google Scholar | With **all** the words – (suicide bereavement) |
|  | With the **exact phrase** – |
|  | With **at least one** of the words – (qualitative research methods interview ethnographic phenomenological experience experiences) |
|  | **Without** the words – |
|  | Where my words occur – ⦿ in the title of the article |
|  | Language = English \| Include citations 🗸 |
